# Endoscopic Surgical Strategy for Upper Lumbar Disc Herniation With Spinal Canal Stenosis: A Technical Note

**DOI:** 10.7759/cureus.96854

**Published:** 2025-11-14

**Authors:** Alisonkenji Kojima, Masahiko Akiyama, Daichi Kawamura, Satoshi Yamana, Yosuke Nakayama, Yuichi Murayama, Hiroki Ohashi

**Affiliations:** 1 Department of Neurosurgery, The Jikei University Hospital, Tokyo, JPN; 2 Department of Neurosurgery, Sapporo Teishinkai Hospital, Sapporo, JPN; 3 Department of Neurosurgery, The Jikei University Hospital,, Tokyo, JPN

**Keywords:** full-endoscopic lumbar discectomy, full-endoscopic spine surgery, interlaminar approach, lumbar spinal canal stenosis, upper lumbar disc herniation

## Abstract

Treatment of upper lumbar disc herniation (LDH) with concomitant spinal canal stenosis is challenging. Conventionally, addressing both pathologies endoscopically requires the use of both transforaminal and interlaminar endoscopic systems, which increases surgical invasiveness and procedural complexity. In this study, we adopted a novel technique involving full endoscopic lumbar laminectomy via an interlaminar approach, followed by the application of a specially designed retraction tube. This method allowed safe and minimally invasive removal of central-type upper LDH from both sides without requiring endoscope exchange. In this article, we describe the technical aspects of endoscopic spinal surgery for patients with upper LDH and lumbar spinal canal stenosis.

## Introduction

Upper lumbar disc herniation (LDH) refers to the rupture of the fibrous annulus and protrusion of the nucleus pulposus at L3-4 or above, with a low incidence of 1% to 10.4% [1-3].

Compared with lower LDH, upper LDH has unique anatomical characteristics, including a narrower spinal canal, reduced distance between nerve roots and dura mater, shorter nerve roots in the intervertebral foramen, and proximity to the conus medullaris. These features make surgery more challenging.

Clinically, patients with upper LDH often experience severe back and leg pain, muscle weakness, and gait disturbance, which can significantly impair quality of life. Conventional open surgery is effective but may cause greater muscle damage, postoperative pain, and longer recovery times.

With advancements in spinal endoscopic techniques, their indications have progressively expanded. Recently, endoscopic surgery for upper LDH has demonstrated clinical outcomes comparable to those of conventional open surgery [4]. However, the presence of spinal canal stenosis markedly increases the technical difficulty of endoscopic procedures. In such cases, we previously performed transforaminal full endoscopic discectomy followed by interlaminar full endoscopic laminectomy, or simultaneously, using two different endoscopic systems. However, this method introduces practical challenges, including equipment complexity, prolonged operative time, and increased patient burden.

In this study, we first performed a laminectomy using a stenosis-specific endoscope via an interlaminar approach. Subsequently, by utilizing a dedicated retraction tube, we achieved bilateral, single-session removal of central-type upper LDH without the need to exchange the endoscope. This technical note aims to describe a simplified single-endoscope method for treating patients with upper LDH and spinal stenosis, reducing invasiveness and operative time in detail.

## Technical report

**Figure 1:**
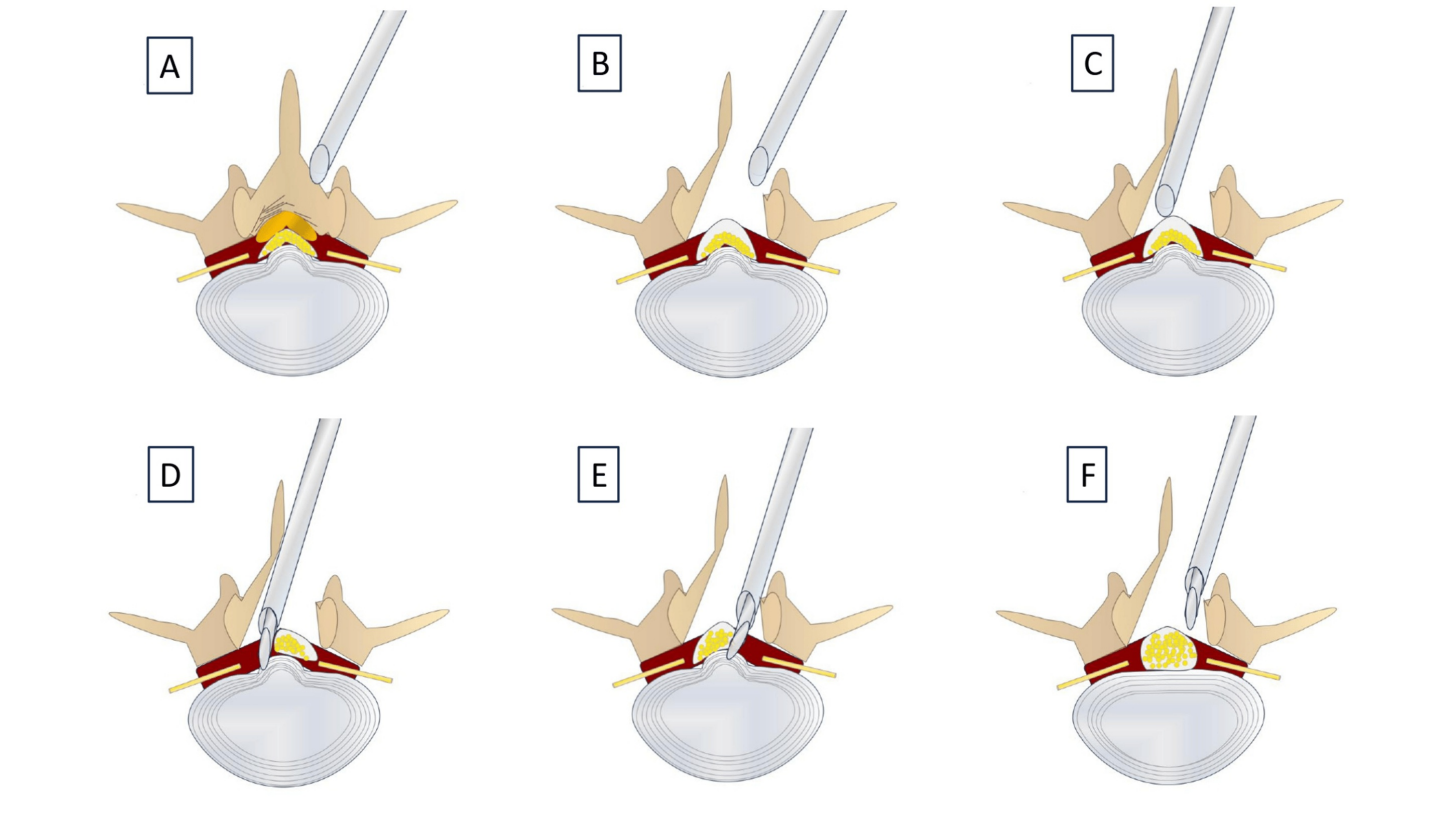
Step-by-step instructions for endoscopic lumbar laminectomy and discectomy. (A) Identification of the base of the spinous process in the cranial vertebral lamina. (B) Laminectomy and ligamentum flavum resection. (C) The endoscope was oriented toward the contralateral side of the dura. An Elliquence retraction tube was inserted, and the dura was gently retracted. (D) Discectomy was performed using forceps inserted through the retraction tube channel. (E) Discectomy was performed on the ipsilateral side. (F) Completion of a full endoscopic lumbar laminectomy and discectomy using a single scope in a single surgical session. Image credit: All authors.

Step-by-step instructions are illustrated in Figure 1.

Position and Skin Incision

**Figure 2:**
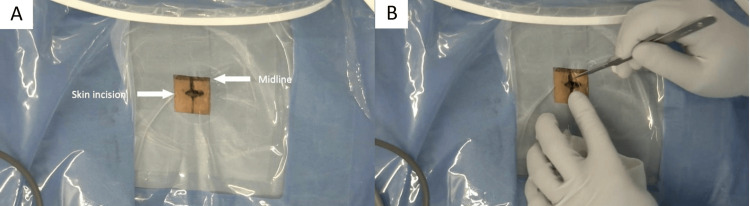
Skin Incision. (A) A 12-mm skin incision was made 10 mm lateral to the midline at the target vertebral level under fluoroscopic guidance. (B) The length of the skin incision was comparable to that of the index fingernail.

The patient was placed in the prone position, and surgery was performed under general anesthesia combined with motor-evoked potential monitoring. A 12-mm skin incision was made 10 mm lateral to the midline at the target vertebral level under fluoroscopic guidance (Figure 2).

Exposure, Laminectomy, and Removal of Ligamentum Flavum

Using a dilator, the muscle attached between the inferior margin of the cranial vertebral lamina (VL) and the superior margin of the caudal VL was gently detached. Subsequently, an angled working sheath and a working channel endoscope (7.1-mm diameter) were inserted. A specialized retraction tube (Elliquence, Baldwin, NY) was introduced along with bipolar cautery (Trigger-Flex® bipolar probe, Elliquence) and a micro punch to remove soft tissue and expose the VL.

**Figure 3:**
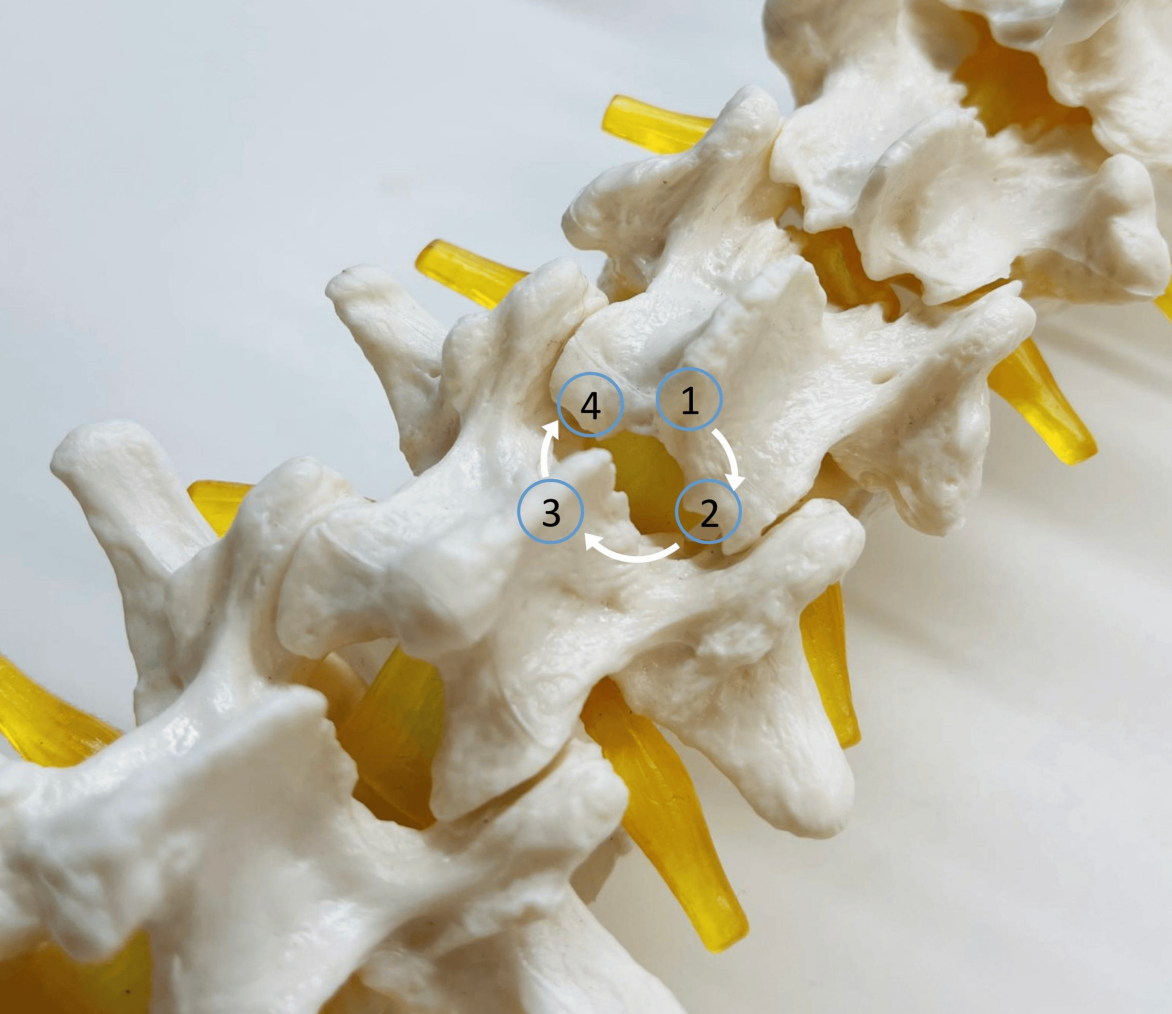
Bone removal was performed in the following order: ① inferior margin of the cranial lamina, ② ipsilateral lateral side, ③ caudal lamina, and ④ contralateral lateral side.

Bone removal was performed using a high-speed drill (Primado2, Nakanishi Inc., Tochigi, Japan) (Figure 3). The base of the spinous process was identified, and bone removal commenced from its lower margin at the upper VL. Hemostasis of bleeding from cancellous bone was effectively achieved using bipolar cautery and bone wax. To excise the ligamentum flavum as a single piece whenever possible, bone was removed carefully by identifying and following its attachment sites until it could be safely detached.

**Figure 4:**
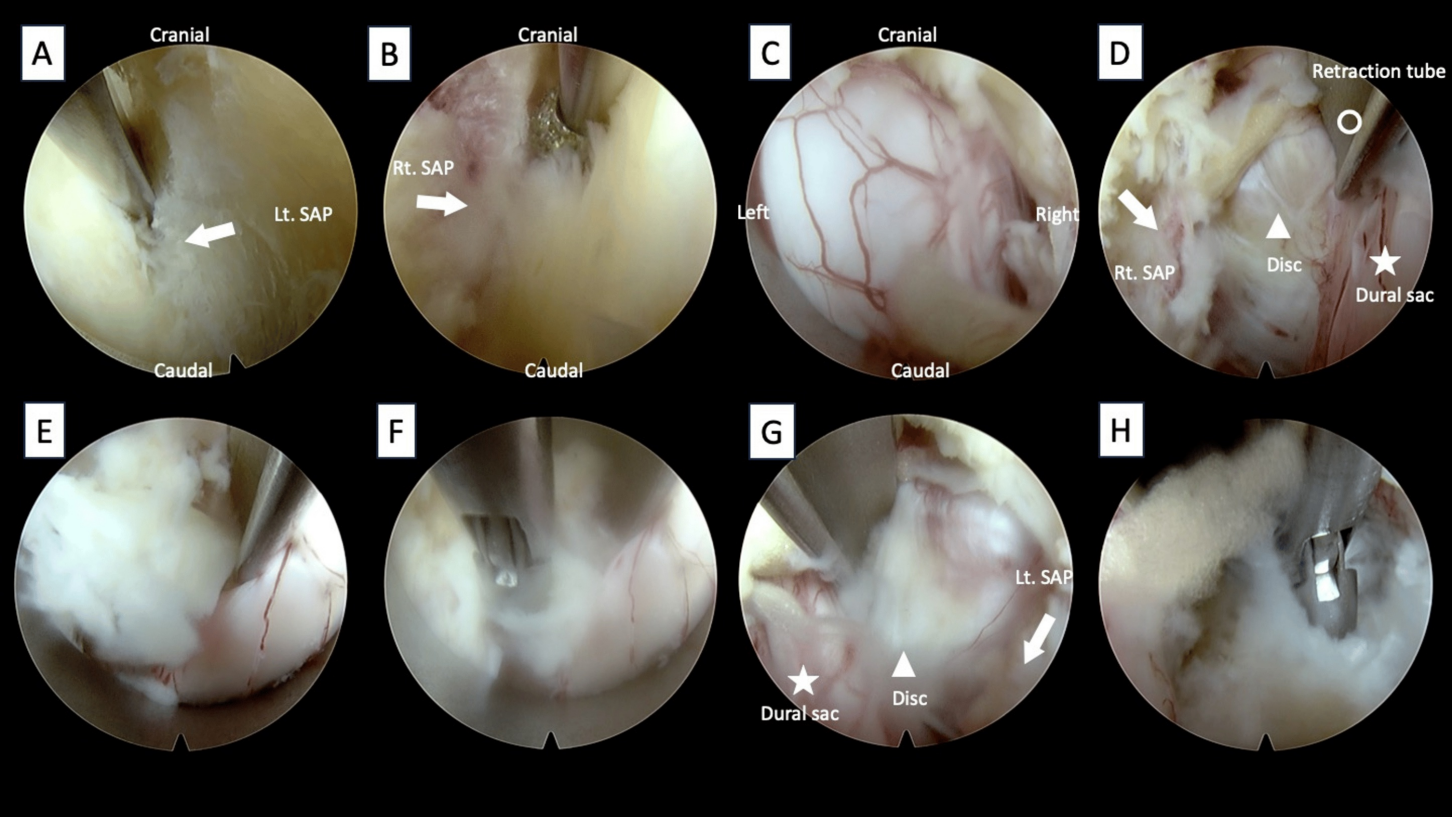
Intraoperative images illustrating sequential surgical steps. (A) Confirmation of the extent of lateral bone removal on the ipsilateral side. (B) Confirmation of the extent of lateral bone removal on the contralateral side. (C) Sufficient exposure of the dural sac following ligamentum flavum removal. (D) Retraction of the dural sac(asterisk) with the tip of the Elliquence retraction tube (open circle), exposing the herniated disc (arrowhead). (E) Extruded disc. (F) Removal of the extruded disc with forceps. (G) Retraction of the dural sac (asterisk) from the contralateral side, exposing the herniated disc (arrowhead). (H) Removal of the herniated disc from the ipsilateral side.

The endoscope was then oriented toward the ipsilateral side, and lateral bone removal was advanced. Under intraoperative fluoroscopic guidance, decompression was confirmed to have reached the medial border of the superior articular process (SAP) (Figure 4A).

Thereafter, the upper margins of the lower laminae were excised. Finally, the contralateral bone was removed. During this step, bone resection was extended laterally to the attachment of the ligamentum flavum at the SAP to complete the laminectomy (Figure 4B).

Discectomy

**Figure 5:**
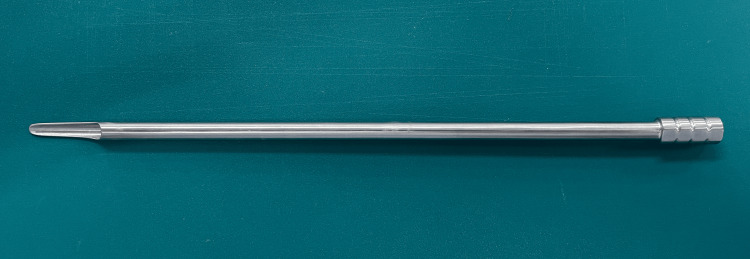
A specially designed shoehorn-shaped retraction tube was used in this procedure. It has an outer diameter of 7.0 mm and is available in lengths of 250 mm and 280 mm. The distal end is spatula-shaped, and the shaft is hollow to accommodate surgical instruments.

Following resection of the ligamentum flavum and sufficient exposure of the dura mater (Figure 4C), the endoscope was directed toward the lateral aspect of the dura. Using a specially designed, shoehorn-shaped retraction tube (Elliquence) (Figure 5), the dura was gently retracted to expose the intervertebral disc (Figure 4D). Excessive retraction was avoided because of the close proximity of the conus medullaris. While retracting the dura with the tip of the retraction tube, its hollow structure allowed insertion of forceps through the tube, enabling adequate removal of the herniated disc (Figures 4E, 4F). Because sufficient lateral laminectomy had been performed, the same procedure was repeated on the contralateral side (Figures 4G, 4H). Ultimately, bilateral maximal removal of the intervertebral discs was achieved, resulting in adequate decompression of the dura mater.

Wound Closure and Postoperative Care

We prefer placing a drain through the portal to avoid the risk of epidural hematoma. The application of skin glue over one or two subcutaneous sutures is sufficient for wound closure.

Case 1

**Figure 6:**
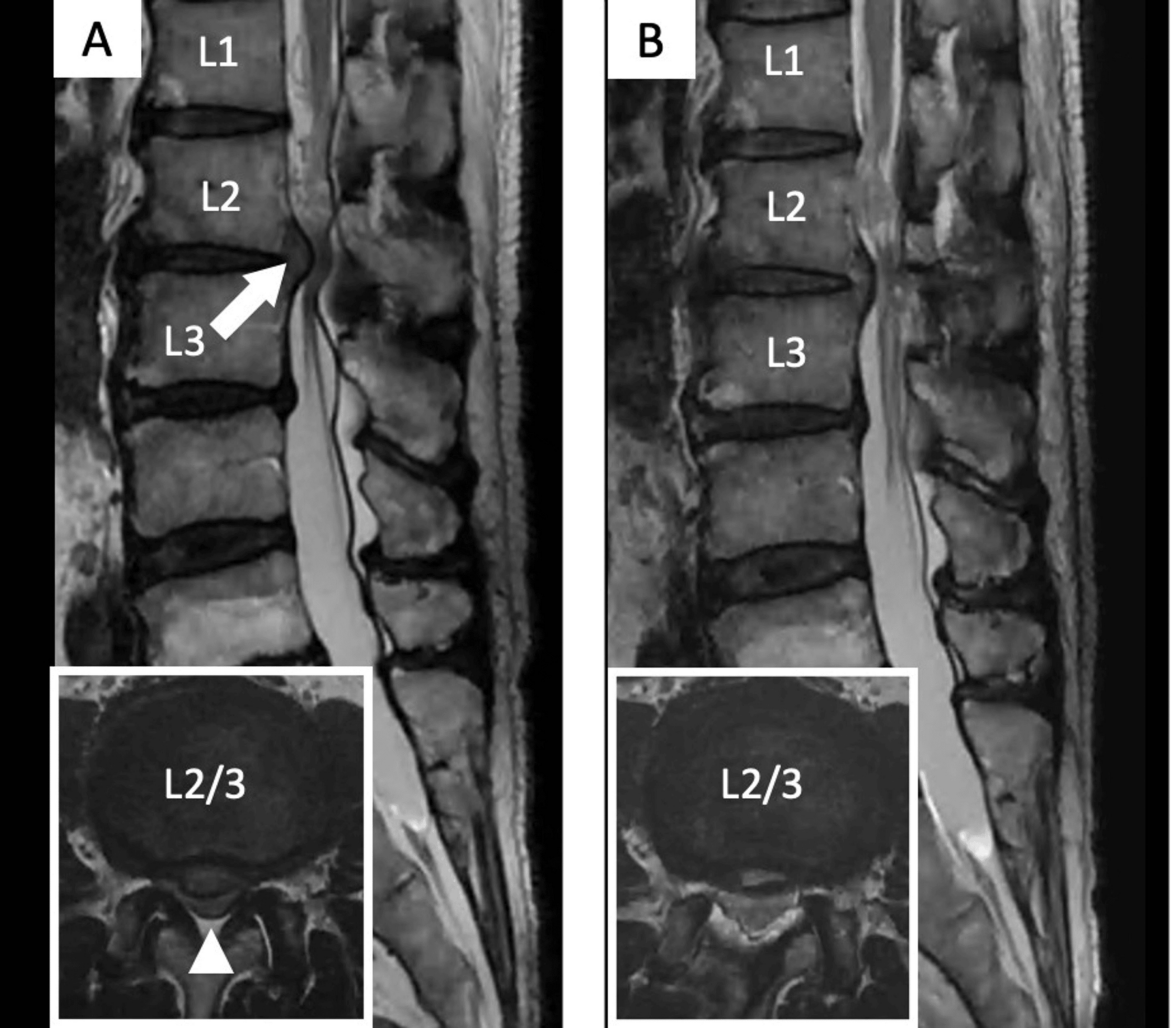
Case 1: Preoperative and postoperative magnetic resonance imaging (MRI). (A) Preoperative MRI reveals a central-type disc herniation (arrow) with spinal canal stenosis (arrowhead) compressing the dural sac at L2–L3. (B) Postoperative MRI shows complete laminectomy and adequate removal of the herniated disc.

A 62-year-old male with a one-year history of back pain and radiating discomfort in both lower extremities underwent magnetic resonance imaging, which revealed a central-type LDH at the level of L2-3 with spinal canal stenosis (Figure 6A). Endoscopic laminectomy and discectomy were performed via the interlaminar approach.

Postoperative magnetic resonance imaging revealed a successful laminectomy and removal of the herniated disc, with expansion of the dural sac observed (Figure 6B). No surgery-related complications were observed, and the symptoms improved promptly after the operation. At the one-year follow-up, the lumbar Japanese Orthopedic Association (JOA) score improved from 16 preoperatively to 28, and no recurrence of symptoms was noted.

## Discussion

Anatomical features of the upper lumbar vertebral bodies and their associated structures differ considerably from those of the lower lumbar spine. Vertebral bodies and intervertebral discs in the upper lumbar region are relatively small, and the spinal canal typically assumes an oval configuration with absent or shallow lateral recesses. The epidural space is notably narrow and contains minimal epidural fat, thereby providing limited buffering capacity around the dural sac. Moreover, a greater density of neural elements is present within the dura in this region, and the nerve roots are characteristically short and run predominantly horizontally [5,6].

Therefore, once a disc herniation occurs, even if mild, it can cause significant compression of the spinal cord, resulting in corresponding neurological symptoms. Disc herniation in this region does not directly compress a single nerve root but rather compresses the dural tissue, causing complex and diverse clinical manifestations [7].

When upper LDH occurs and conservative treatment fails, traditional surgical methods are employed, which include lumbar microdiscectomy and lumbar discectomy combined with intervertebral fusion [8]. With recent advances in surgical techniques, full endoscopic spinal surgery has become widely adopted as a minimally invasive technique and has proven to be safe and feasible. Full endoscopic transforaminal discectomy is reported to be an appropriate and viable choice for patients with upper LDH without dural traction or the need for laminectomy, demonstrating clinical outcomes comparable to those achieved with conventional open surgery [9,10]. Transforaminal unilateral biportal endoscopic discectomy has also been reported as an effective approach for upper LDH, offering favorable surgical outcomes [11].

However, when patients with upper LDH also present with spinal canal stenosis, treatment becomes even more challenging. We encountered two consecutive cases in which patients with upper LDH complicated by spinal canal stenosis initially underwent full-endoscopic lumbar discectomy, which resulted in symptomatic improvement (Cases 2 and 3).

The clinical characteristics and postoperative outcomes of these cases are summarized in Table 1.

**Table 1:** Clinical summary of three cases and postoperative changes in JOA score. FELD, full-endoscopic lumbar discectomy; FELL, full-endoscopic lumbar laminectomy; JOA, Japanese Orthopedic Association (maximum score: 29 points)

	Case 1	Case 2	Case 3
Age (years)	62	75	66
Sex	Male	Male	Male
Level of pathology	L2/3	L1/2	L1/2
Operative time (minutes)	150 (FELD + FELL)	36 (FELD), 115 (FELL)	43 (FELD), 130 (FELL)
JOA score (preoperative)	16	2	21
JOA score (six months postoperative)	27	11	12
JOA score (one year postoperative)	28	17	24
Postoperative course	No procedural complication	No procedural complication	No procedural complication
Time to return to activity (months)	1	11	11

**Figure 7:**
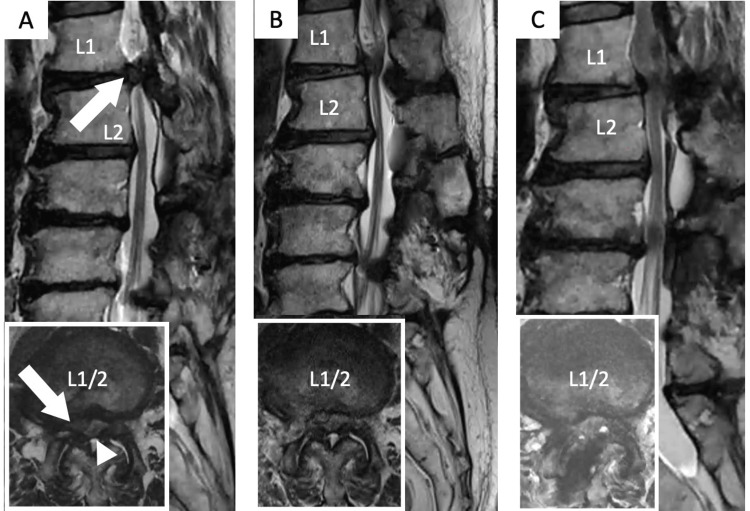
Case 2: A 75-year-old male with intermittent claudication and lower-extremity pain who underwent surgery twice. (A) Preoperative MRI revealing a posterolateral type disc herniation (arrow) with spinal canal stenosis (arrowhead) compressing the dural sac at L1–L2. (B) MRI after full endoscopic lumbar discectomy from the right side via transforaminal approach. (C) MRI after additional full endoscopic lumbar laminectomy from the right side via interlaminar approach (This case is original and has not been previously reported.)

**Figure 8:**
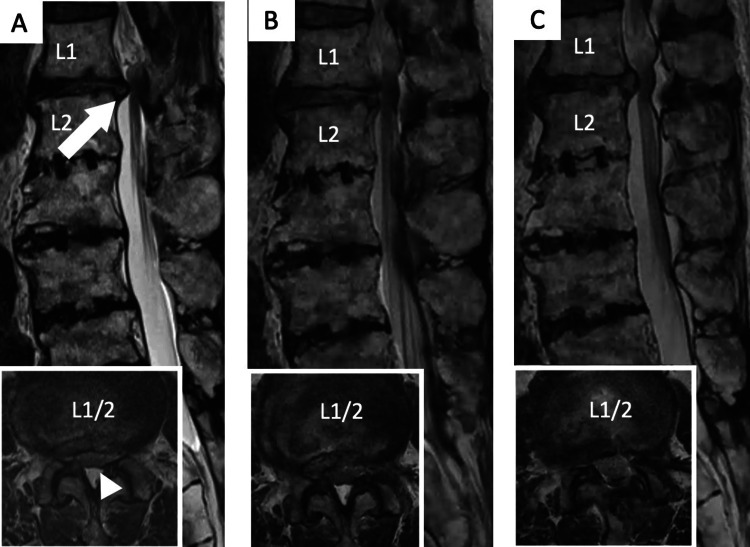
Case 3: A 65-year-old male with radiating pain in his lower extremities, predominantly on the left side. (A) Preoperative MRI revealing a central-type disc herniation (arrow) with spinal canal stenosis (arrowhead) compressing the dural sac at L1–L2. (B) MRI after full endoscopic lumbar discectomy from the left side via transforaminal approach. (C) MRI after additional full endoscopic lumbar laminectomy from the left side via the interlaminar approach. (This case is original and has not been previously reported.)

However, both patients experienced symptom recurrence at three and six months postoperatively, ultimately requiring full-endoscopic lumbar laminectomy via the interlaminar approach (Figures 7, 8). These clinical experiences suggest that in such cases, both disc herniation and spinal canal stenosis should be addressed concurrently.

Traditionally, open surgery has been the standard surgical option in such cases. However, this technique is relatively invasive and often requires additional fusion procedures. Full endoscopic surgery has been increasingly applied in pursuit of minimally invasive treatment; however, full endoscopic discectomy via the transforaminal approach alone, as previously reported, remains insufficient in cases complicated by spinal canal stenosis. Consequently, additional laminectomy via the interlaminar approach becomes necessary. This results in a two-stage procedure, which inevitably increases overall surgical invasiveness.

Complete treatment with a single full endoscopic procedure typically requires the simultaneous use of both transforaminal and interlaminar endoscopic systems. However, this introduces practical challenges, including equipment complexity, prolonged operative time, and increased patient burden.

To overcome these challenges, we employed a specially designed retraction tube (Figure 5). This retraction tube is available in lengths of 250 and 280 mm and has an outer diameter of 7 mm. Although it cannot be inserted through conventional transforaminal stenosis endoscopes, it is compatible with the Elliquence Stenosis Scope, which has a 7.1-mm working channel. The tip of the instrument is shaped like a spatula, and the shaft is hollow, enabling it to safely retract the dura with its tip while allowing simultaneous insertion of forceps and other instruments for discectomy.

Although the use of a large-diameter stenosis endoscope to retract the dura directly via its outer sheath during interlaminar laminectomy poses a risk, this specialized retraction tube allows for safe and adequate bilateral discectomy. This technique does not require advanced skills or specialized technology and is an effective method for treating both spinal canal stenosis and upper LDH through a single skin incision using a single endoscope in a single surgical session.

## Conclusions

The application of a specially designed shoehorn-shaped retraction appears feasible and may be effective in full endoscopic surgery for carefully selected patients with upper LDH and spinal canal stenosis. This method enables complete treatment of upper LDH with spinal canal stenosis through a single skin incision, with a single endoscope, in a single surgical session, and may serve as a promising minimally invasive alternative.

This technical note has several limitations: the sample size is small, long-term follow-up is lacking, and no systematic comparative analysis has been performed. Therefore, the findings should be regarded as preliminary. Further studies with a larger number of cases, longer follow-up, and comparative analyses are warranted.
